# Supplementary material for: Plant Leaf Functional Adaptions along Urban–Rural Gradients of Jinhua City
Source: Plants (Basel). 2024 Jun 7;13(12):1586. doi: 10.3390/plants13121586 (PMC11207212; doi:10.3390/plants13121586)
Supplement: Supplementary file 1 [file plants-13-01586-s001.zip › plants-2925788-supplementary.pdf]

Supplementary Information

Plant Leaf Functional Adaptions along Urban–Rural Gradients of Jinhua City

Chenchen Cao [1,†], Shufen Cui [2,†], Xinyu Guan [1], Yuanjian Chen [1], Yongqi Zhang [1], Xingwen Lin [1], Chaofan Wu [1], Zhaoyang Zhang [1], Fei Zhang [1], Yuling Xu [3] and Zhenzhen Zhang [1,*]

[1] College of Geography and Environmental Sciences, Zhejiang Normal University, Jinhua 321004, China; caochenchen@zjnu.edu.cn (C.C.); 202120200822@zjnu.edu.cn (X.G.); crossborder@zjnu.edu.cn (Y.C.); zhang-yongqi123@zjnu.edu.cn (Y.Z.); linxw@zjnu.edu.cn (X.L.); cfwdh@zjnu.edu.cn (C.W.); zhzhyang@outlook.com (Z.Z.); zhangfei3s@163.com (F.Z.)
[2] College of Business, Lishui University, Lishui 323200, China; 471101875@lsu.edu.cn
[3] Zhejiang Jinhua Ecological and Environmental Monitoring Center, Jinhua 321000, China; xuyul0906@gmail.com
* Correspondence: zhangzhen@zjnu.cn
† These authors contributed equally to this work.

Table S1 Three-way ANOVA of Plant Functional Traits Across an Urbanization Gradient.

Traits	Sample number	Urbanization gradients			Tree type			Time			Urbanization gradients×Tree type			Urbanization gradients×Time			Time×Tree type			Urbanization gradients×Time×Tree type		
		df	P	F	df	P	F	df	P	F	df	P	F	df	P	F	df	P	F	df	P	F
LWC	450	2	0.081	2.528	1	0.000**	89.365	1	0.000**	25.482	2	0.093	2.392	2	0.007**	4.973	1	0.018*	5.677	2	0.788	0.238
LDMC	450	2	0.081	2.528	1	0.000**	89.365	1	0.000**	25.482	2	0.093	2.392	2	0.007**	4.973	1	0.018*	5.677	2	0.788	0.238
WD	450	2	0.333	1.104	1	0.031	4.697	1	0.000**	22.915	2	0.037*	3.322	2	0.011*	4.555	1	0.236	1.407	2	0.324	1.129
Hv	450	2	0.292	1.233	1	0.000**	23.756	1	0.000**	3.217	2	0.049*	3.038	2	0.855	0.156	1	0.929	0.008	2	0.363	1.016
LT	450	2	0.002**	6.362	1	0.000**	87.644	1	0.000**	0.524	2	0.244	1.416	2	0.393	0.935	1	0.266	1.241	2	0.766	0.266
SLA	450	2	0.002**	6.553	1	0.000**	258.266	1	0.603	32.523	2	0.017*	4.118	2	0.27	1.314	1	0.015*	5.934	2	0.711	0.341
LTD	450	2	0.000**	20.065	1	0.000**	16.493	1	0.000**	50.715	2	0.043*	3.179	2	0.003**	5.741	1	0.204	1.616	2	0.735	0.308
Chl	450	2	0.033*	3.442	1	0.000**	88.782	1	0.000**	9.65	2	0.33	1.112	2	0.077	2.586	1	0.003**	9.098	2	0.758	0.277
SD	450	2	0.131	2.043	1	0.000**	21.174	1	0.000**	0.41	2	0.432	0.84	2	0.638	0.45	1	0.722	0.127	2	0.747	0.292
SS	450	2	0.000**	7.604	1	0.000**	56.046	1	0.000**	5.135	2	0.000**	7.929	2	0.784	0.243	1	0.245	1.352	2	0.588	0.532

*p<=0.05 ** p<=0.01

Table S2 One-way ANOVA of plant functional traits of deciduous species *Prunus subg. Cerasus* sp and *Liquidambar formosana* on urban-rural gradient

	Prunus subg. Cerasus sp (Deciduous)			*Liquidambar formosana* (Deciduous)		
	Urban	Surburb	Rural	Urban	Surburb	Rural
LWC	61.23 ± 3.25	63.64 ± 2.64	64.98 ± 5.5	58.22 ± 2.2	60.68 ± 4.62	66.1 ± 8.82
LDMC	38.77 ± 3.25	36.36 ± 2.64	35.02 ± 5.5	41.78 ± 2.2	39.32 ± 4.62	33.9 ± 8.82
WD	0.58 ± 0.1	0.55 ± 0.11	0.51 ± 0.07	0.5 ± 0.06	0.49 ± 0.05	0.44 ± 0.11
Hv	0.02 ± 0.01	0.02 ± 0.01	0.02 ± 0.01	0.01 ± 0.01	0.02 ± 0.01	0.02 ± 0.01
LT	**0.18 ± 0.01b**	**0.12 ± 0.01c**	**0.2 ± 0.02b**	0.2 ± 0.02	0.21 ± 0.03	0.2 ± 0.01
SLA	139.67 ± 17.59	148.6 ± 11.4	153.93 ± 27.13	129.39 ± 18.58	125.7 ± 33.14	139.95 ± 7.82
LTD	**0.3± 0.05b**	**0.4 ± 0.06a**	**0.33 ± 0.04b**	**0.3 ± 0.06b**	**0.4 ± 0.03a**	**0.29 ± 0.02b**
Chl	45.95 ± 3.84	43.85 ± 3.09	43.67 ± 2.89	**34.43 ± 3.19b**	**42.36 ± 8.06a**	**26.58 ± 0.77c**
SD	**62.52 ± 12.08ab**	**60.46 ± 14.99ab**	**51.67 ± 8.72b**	148.44 ± 21.15	135.63 ± 24.82	165.82 ± 7.29
SS	**2.53 ± 0.24b**	**3.19 ± 0.51a**	**3.15 ± 0.51a**	**4.29 ± 0.54bc**	**2.32 ± 0.25d**	**3.27 ± 0.45cd**

Different lowercase letters indicated significant differences in plant functional traits on urban and rural gradients ($p < 0.05$).

Table S3 One-way ANOVA of plant functional traits of evergreen species *Cinnamomum camphora* and *Photinia serrulata* Lindl. on urban-rural gradient

	Cinnamomum camphora (Evergreen)			*Photinia serrulata* Lindl. (Evergreen)		
	Urban	Surburb	Rural	Urban	Surburb	Rural
LWC	56.84 ± 2.48	55.44 ± 1.57	57.24 ± 7.41	**59.99 ± 1.99a**	**56.38 ± 1.11b**	**55.62 ± 1.15b**
LDMC	43.16 ± 2.48	44.56 ± 1.57	42.76 ± 7.41	**40.01 ± 1.99b**	**43.62 ± 1.11a**	**44.38 ± 1.15a**
WD	0.45 ± 0.12	0.41 ± 0.04	0.37 ± 0.08	0.73 ± 0.21	0.55 ± 0.04	0.55 ± 0.09
Hv	0.02 ± 0.01	0.02 ± 0.01	0.03 ± 0.01	0.02 ± 0	0.02 ± 0.01	0.03 ± 0.01
LT	0.19 ± 0.02	0.19 ± 0.02	0.18 ± 0.04	0.28 ± 0.03	0.3 ± 0.01	0.29 ± 0.04
SLA	122.77 ± 12.12	112.7 ± 5.46	127.15 ± 17.09	**89.48 ± 4.71a**	**74.07 ± 6.85b**	**81.92 ± 8.51ab**
LTD	0.43 ± 0.05	0.46 ± 0.03	0.45 ± 0.1	0.4 ± 0.06	0.37 ± 0.04	0.43 ± 0.03
Chl	**35.96 ± 5.52b**	**43.51 ± 2.24a**	**38.59 ± 4.03ab**	54.35 ± 7.94	55.45 ± 4.82	49.4 ± 4.1
SD	73.36 ± 10.29	81.58 ± 7.34	74.77 ± 10.79	92 ± 10.12	76.61 ± 13.97	92.5 ± 6.37
SS	**4.46 ± 0.59b**	**5.77 ± 0.37a**	**5.32 ± 1.17ab**	5.24 ± 2.24	3.22 ± 0.49	3.39 ± 0.66

Different lowercase letters indicated significant differences in plant functional traits on urban and rural gradients ($p < 0.05$).

Table S4 One-way ANOVA of plant functional traits of evergreen species *Osmanthus fragrans* (Thunb.) Loureiro and *Ligustrum lucidum* Ait on urban-rural gradient

	Osmanthus fragrans (Thunb.) Loureiro (Evergreen)			*Ligustrum lucidum* Ait (Evergreen)		
	Urban	Surburb	Rural	Urban	Surburb	Rural
LWC	46.91 ± 5.21	47.92 ± 5.32	50.47 ± 3.84	64 ± 2.01	64.88 ± 4.8	65.46 ± 4.94
LDMC	53.09 ± 5.21	52.08 ± 5.32	49.53 ± 3.84	36 ± 2.01	35.12 ± 4.8	34.54 ± 4.94
WD	**0.61 ± 0.1a**	**0.43 ± 0.1b**	**0.55 ± 0.08a**	0.42 ± 0.14	0.54 ± 0.08	0.45 ± 0.1
Hv	0.01 ± 0.01	0.01 ± 0.01	0.02 ± 0.01	0.02 ± 0.01	0.01 ± 0.01	0.02 ± 0.01
LT	0.26 ± 0.01	0.27 ± 0.01	0.25 ± 0.03	**0.31 ± 0.03a**	**0.26 ± 0.04ab**	**0.25 ± 0.02b**
SLA	74.16 ± 11.74	71.19 ± 6.4	79.64 ± 9.88	93.19 ± 9.56	123.2 ± 22.21	108.33 ± 41.15
LTD	0.54 ± 0.08	0.53 ± 0.06	0.53 ± 0.08	0.35 ± 0.05	0.32 ± 0.04	0.42 ± 0.22
Chl	44.75 ± 8.29	40.77 ± 6.06	45.15 ± 6.01	59.79 ± 6.76	57.33 ± 6.33	59.6 ± 5.32
SD	**149.08 ± 21.09b**	**164.18 ± 10.4ab**	**175.97 ± 33.74a**	88.84 ± 38.7	58.53 ± 2.59	59.17 ± 8.78
SS	**7.03 ± 0.95a**	**5.1 ± 0.88b**	**6.33 ± 1.07a**	4.78 ± 1	5.16 ± 0.9	4.83 ± 0.41

Different lowercase letters indicated significant differences in plant functional traits on urban and rural gradients ($p < 0.05$).

Table S5 One-way ANOVA of changes in functional traits of deciduous species *Prunus subg. Cerasus* sp and *Liquidambar formosana* from June to August on urban-rural gradient

	Prunus subg. Cerasus sp (Deciduous)			*Liquidambar formosana* (Deciduous)		
	Urban	Surburb	Rural	Urban	Surburb	Rural
LWC	**-2.13 ± 1.72ab**	**-2.4 ± 2.41b**	**-7.4 ± 1.15c**	**-1.8 ± 1.89ab**	**0.94 ± 3.09b**	**-12.58 ± 5.22c**
LDMC	**2.13 ± 1.72bc**	**2.4 ± 2.41b**	**7.4 ± 1.15a**	**1.8 ± 1.89bc**	**-0.94 ± 3.09b**	**12.58 ± 5.22a**
WD	**0.02 ± 0.03b**	**0.04 ± 0.03ab**	**0.06 ± 0.02a**	0.01 ± 0.03	0.01 ± 0.05	0.15 ± 0.05
Hv	**-0 .003± 0.001b**	**0.01 ± 0.005a**	**0.002 ± 0.003ab**	**-0.001 ± 0.001b**	**0.008 ± 0.04a**	**0.003 ± 0.01ab**
LT	-0.01 ± 0.01	-0.02 ± 0.03	-0.01 ± 0.01	-0.01 ± 0.01	-0.02 ± 0.02	-0.01 ± 0.01
SLA	-22.83 ± 9.69	-30.58 ± 12.91	-27.63 ± 9.97	-25.54 ± 4.79	-30 ± 4.57	-31.58 ± 7.67
LTD	0.07 ± 0.03	0.23 ± 0.11	0.17 ± 0.02	**0.02 ± 0.02c**	**0.21 ± 0.02a**	**0.12 ± 0.02b**
Chl	**-4.27 ± 1.2c**	**-3.01 ± 3.59bc**	**2.51 ± 2.15ab**	-1.76 ± 1.44	-0.61 ± 4.8	2.17 ± 1.07
SD	**4.43 ± 2.49a**	**5.53 ± 6.47a**	**-10.99 ± 4.16b**	**10 ± 4.67a**	**8.53 ± 4.36a**	**-8.4 ± 12.57b**
SS	**0.91 ± 0.29a**	**-0.33 ± 0.39b**	**0.57 ± 0.3ab**	**0.71 ± 0.2a**	**-0.21 ± 0.11b**	**0.5 ± 0.42ab**

Different lowercase letters indicated significant differences in plant functional traits on urban and rural gradients ($p < 0.05$).

Table S6 One-way ANOVA of changes in functional traits of evergreen species *Cinnamomum camphora* and *Photinia serrulata* Lindl. from June to August on urban-rural gradient

	Cinnamomum camphora (Evergreen)			*Photinia serrulata* Lindl. (Evergreen)		
	Urban	Surburb	Rural	Urban	Surburb	Rural
LWC	**-0.36 ± 0.73a**	**-2.43 ± 2.14ab**	**-5.99 ± 2.39b**	**-1.03 ± 0.91a**	**-2.65 ± 2.17ab**	**-4.89 ± 0.78b**
LDMC	**0.36 ± 0.73c**	**2.43 ± 2.14bc**	**5.99 ± 2.39b**	**1.03 ± 0.91c**	**2.65 ± 2.17bc**	**4.89 ± 0.78b**
WD	0.02 ± 0.03	0.05 ± 0.02	0.04 ± 0.01	0.12 ± 0.16	0.08 ± 0.06	0.04 ± 0.04
Hv	0.001 ± 0.001	0.001 ± 0.002	0.002 ± 0.001	0.003 ± 0.001	0.001 ± 0.002	0.002 ± 0.01
LT	**0.01 ± 0.01a**	**-0.04 ± 0.03b**	**0.02 ± 0.01a**	**0.02 ± 0.01a**	**-0.03 ± 0.02b**	**0.02 ± 0.01a**
SLA	**-11.12 ± 2.92b**	**-5.78 ± 5.87a**	**-13.02 ± 4.64b**	-20.09 ± 0.54	-9.52 ± 5.42	-12.71 ± 2.78
LTD	**0.01 ± 0.02b**	**0.15 ± 0.01a**	**0.03 ± 0.03b**	0.02 ± 0.01	0.03 ± 0.02	0.03 ± 0.01
Chl	4.79 ± 1.18	2.42 ± 3.69	6.44 ± 1.84	**5.18 ± 2.87a**	**2.14 ± 1.89b**	**4.23 ± 1.13ab**
SD	**5 ± 3.72a**	**-1.08 ± 5.2b**	**3.12 ± 2.19ab**	**4.23 ± 3.02a**	**-7.39 ± 1.67b**	**2.17 ± 5.6ab**
SS	0.35 ± 0.2	0.6 ± 0.55	0.2 ± 0.37	0.8 ± 0.24	0.52 ± 0.27	0.75 ± 0.37

Different lowercase letters indicated significant differences in plant functional traits on urban and rural gradients ($p < 0.05$).

Table S7 One-way ANOVA of changes in functional traits of evergreen species *Osmanthus fragrans* (Thunb.) Loureiro and *Ligustrum lucidum* Ait from June to August on urban-rural gradient

	Osmanthus fragrans (Thunb.) Loureiro (Evergreen)			*Ligustrum lucidum* Ait (Evergreen)		
	Urban	Surburb	Rural	Urban	Surburb	Rural
LWC	**-1.14 ± 1.09a**	**-2.34 ± 2.51ab**	**-5.93 ± 3.52b**	**-1.48 ± 1.12a**	**-2.3 ± 2.03ab**	**-4.17 ± 1.31b**
LDMC	**1.14 ± 4.09c**	**2.34 ± 2.51bc**	**5.93 ± 3.52b**	**1.48 ± 1.12c**	**2.3 ± 2.03bc**	**4.17 ± 1.31b**
WD	**0.01 ± 0.03b**	**0.07 ± 0.04a**	**0.02 ± 0.02b**	0.03 ± 0.02	0.05 ± 0.02	0.04 ± 0.02
Hv	0.002 ± 0.001	-0.001 ± 0.002	0.002 ± 0.001	0.003 ± 0.002	0.001 ± 0.001	0.001 ± 0.001
LT	**0.01 ± 0.01a**	**-0.03 ± 0.02b**	**0.01 ± 0.01a**	**0.015 ± 0.01a**	**-0.02 ± 0.02b**	**0.016 ± 0.001a**
SLA	**-4.24 ± 3.16a**	**-5.55 ± 3.2a**	**-13.13 ± 4.11b**	**-3 ± 2.06a**	**-10.87 ± 7.18ab**	**-20.84 ± 7.16b**
LTD	**0.02 ± 0.01b**	**0.16 ± 0.05a**	**0.05 ± 0.02b**	-0.02 ± 0.01	0.05 ± 0.03	0.03 ± 0.01
Chl	**3.03 ± 2.63b**	**3.52 ± 2.45b**	**6.52 ± 2.15a**	3.92 ± 1.28	4.36 ± 2.29	7.3 ± 2.93
SD	**7.55 ± 4.02a**	**-6.15 ± 5.08b**	**3.92 ± 3.17ab**	5.09 ± 2.04	-5.78 ± 2.54	2.28 ± 2.6
SS	0.23 ± 0.17	0.33 ± 0.32	0.41 ± 0.25	0.6 ± 0.2	0.73 ± 0.32	0.62± 0.14

Different lowercase letters indicated significant differences in plant functional traits on urban and rural gradients ($p < 0.05$).

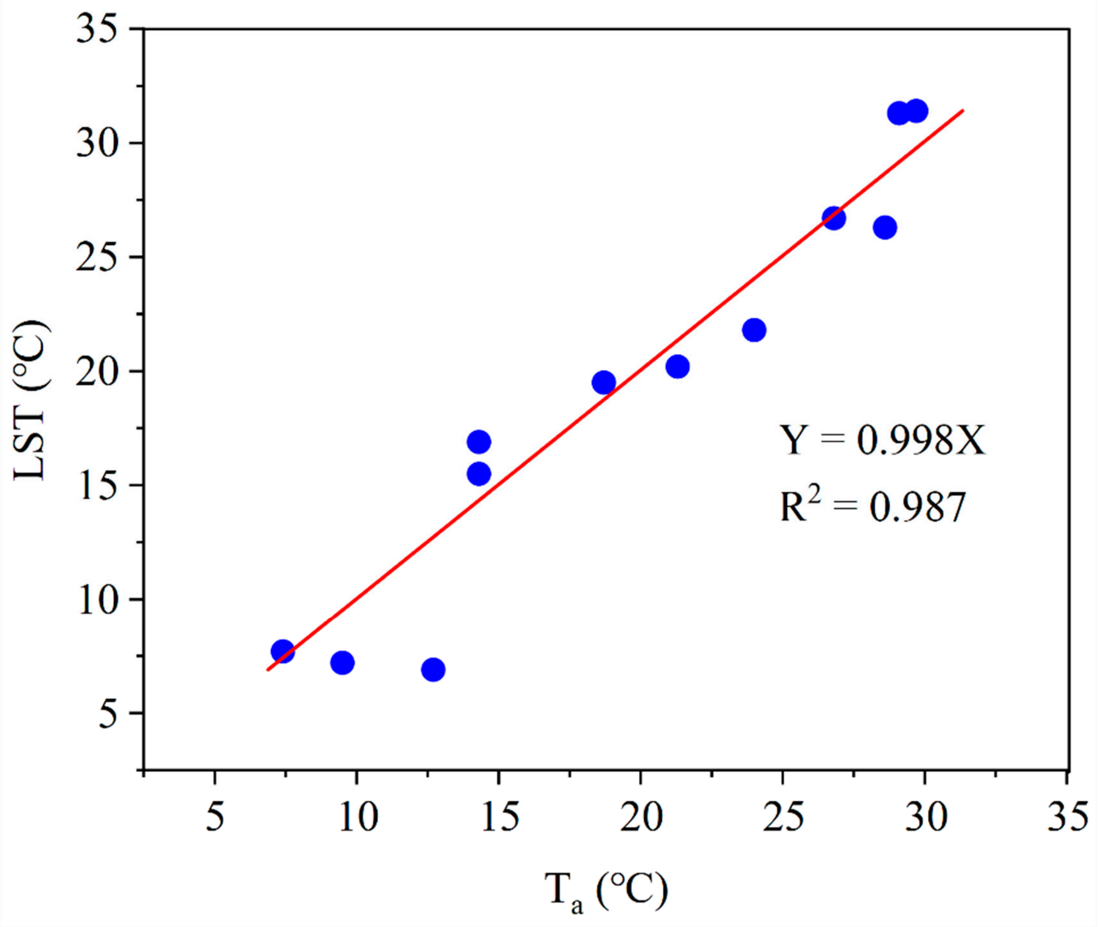

Figure S1 Fitting curve of LST and T_a in Jinhua City in 2022
